# The value of lactate dehydrogenase as a nonspecific tumour marker for seminoma of the testis.

**DOI:** 10.1038/bjc.1982.315

**Published:** 1982-12

**Authors:** A. G. Robertson, G. Read


Br. J. Cancer (1982) 46, 994

*Short Communication*

### THE VALUE OF LACTATE DEHYDROGENASE AS A NONSPECIFIC TUMOUR MARKER FOR SEMINOMA OF THE TESTIS

A. G. ROBERTSON* AND G. READ

*From the Christie Hospital and Holt Radium Institute, Manchester*

Received 5 March 1982 Accepted 24 August 1982

TUMOUR MARKERS have been investigated for over 20 years as a possible means of early diagnosis or assessment of response to treatment. In a number of cases—choriocarcinoma, teratoma, adenocarcinoma of the colon—certain markers have been of value in assessing therapeutic responses and for the detection of tumour recurrence. No marker is yet specific enough to assist in early diagnosis. Teratoma of the testis can produce two markers—human chorionic gonadotrophin (HCG) and Alpha-foetoprotein (AFP); seminoma, on the other hand, is not known to produce a chemically useful marker.

It has been known for many years that serum lactate dehydrogenase (LDH) is elevated in patients with bulky metastatic disease (Brindley & Francis, 1963: Hill & Levi, 1954). This nonspecific marker has not been widely used as it is produced by numerous tumours (Goldman *et al.*, 1964). Recently however it has been suggested that isoenzyme I of LDH may be of value as a marker for testicular tumours (Wampler & Hayra, 1977). In this paper evidence is presented that serum LDH may be of value in identifying patients with advanced seminoma once the diagnosis of seminoma is confirmed, and in assessing the response of patients to treatment.

Thirty-six patients with seminoma of the testis were referred to the Christie Hospital and Holt Radium Institute over an 8-month period. Thirty-two patients had had an orchidectomy performed at another hospital, 2 patients had had a laparotomy and one patient a biopsy of an inguinal node. In one patient the orchidectomy was carried out at the Christie Hospital. This latter patient and one other with advanced disease were seen before surgery. All others were referred after surgery. The pathological material was re-examined in every case. These patients were assessed for the presence of certain tumour markers in their serum. Two further patients who had relapsed after an earlier treatment and who were treated during this period are also included in the study. All patients had chest radiology and i.v. urography. Computer-assisted tomography was performed only in patients in whom there was clinical or radiological suspicion of para-aortic disease.

The staging system used at the Christie Hospital is shown in Table I. Patients with a normal IVU on admission did not have a CAT scan of abdomen, as already stated, nor was lymphangiography carried out on these patients. Patients with minimal involvement of abdominal or pelvic lymph nodes cannot be differentiated from those with no disease. Those with Stage I and Stage IIA disease are therefore grouped together as "early" disease. In 3/27 cases with "early" disease there were tumour cells at the cut end of the cord so that at least 3 patients had Stage IIA disease. The distribution of patients by stage was thus: "early" (including Stage IIA), 27 patients; Stage IIB, 5; Stage III, 3; and Stage IV, 3.

Patients with early disease received

* Present address: Glasgow Institute of Radiotherapeutics and Oncology, Western Infirmary, Glasgow.

TABLE I.—*Christie Hospital: Staging of testicular tumours*

| | | |
|---|---|---|
| I | Disease confined to the testis | "Early" |
| IIA | Abdominal nodes involved but impalpable | |
| IIB | Abdominal nodes involved and palpable | "Late" |
| III | Supradiaphragmatic nodes involved | |
| IV | Extranodal involvement. | |

radiotherapy to the para-aortic and iliac nodes and scrotum. A central midplane dose of 3,000 cGy in 20 fractions over 28 days was given using a 4 or 8 MV linear accelerator Gibb (1960). Patients with Stage IIB disease were treated with a larger abdominal field initially and shielding of at least one kidney was introduced when a central midplane dose of 1,500 cGy was reached. The treatment was then continued to a dose of 3,000 cGy in 4 weeks.

Patients with Stage III or IV disease were assessed individually and treated with either radiation or combination chemotherapy. Patients in whom there was evidence of metastatic disease initially were re-assessed at 3–4 months after treatment using chest radiology and computer-assisted tomography. Further treatment with either radiotherapy or chemotherapy was given if there was residual disease.

All patients with early disease are alive and apparently disease-free. Of the 5 patients with IIB disease 4 were found to be in remission at re-assessment. The remaining patient was found to have a supraclavicular node 6 weeks after radiotherapy in addition to residual abdominal disease. He was treated with combination chemotherapy and is in complete remission.

Of the Stage III patients, 2 received radiotherapy and are in remission, while the third received chemotherapy and is having radiation to residual disease. One Stage IV patient died immediately after radiotherapy, another received chemotherapy and the third received chemotherapy after failure to respond to radiation but has progressive disease.

AFP and HCG were measured by radioimmunoassay. The HCG assays were performed at Charing Cross Hospital and AFP was assayed at the Regional Laboratories in Manchester. The enzymes LDH, $\gamma$-glutamyl transferase (GGT), alkaline phosphatase (Alk P), aspartate transferase (AST) and alanine transferase (ALT) were assayed in the routine biochemistry laboratory at Withington Hospital, Manchester. Ten ml of clotted blood were collected from the patient on admission for radiotherapy before his first treatment. Control levels were those previously established by the Biochemistry Laboratory at Withington. No attempt was made to study the levels of the different isoenzymes of LDH.

The study was partly retrospective. Some assays were not carried out on all of the patients (Table II). Seven markers were evaluated for the detection of bulk disease and, in the case of LDH, for the assessment of response to treatment (Table II).

AFP was elevated in 1/30 cases. This patient had liver metastases. HCG was elevated in 5/31 cases all of whom had advanced disease. Three patients with advanced disease, however, had normal levels. An elevated level did not signify a poor prognosis.

Four enzymes, GGT, Alk P, AST and ALT were also assessed as potential markers. Table (II). Nine of 37 patients had elevated Alk P levels. Two of the 9 cases had early disease. Two patients with advanced disease had normal values. Two of 37 patients had elevated AST levels, one of these had early disease. Nine patients with advanced disease had normal levels. Six of 37 patients had elevated levels of ALT levels. Four of six patients had "early" disease. Two of 38 patients had elevated $\gamma$-GGT levels. Both patients had advanced disease. Nine of 11 patients with advanced disease had normal levels.

Of the 7 markers assessed LDH appeared to be the only one of value. None of the 27 patients with "early" disease had elevated values. Nine of the 11 patients with advanced disease had values out with the normal range (Fig. 1) and one was borderline. The patient with a normal LDH level and advanced disease presented with a recurrence.

In 10/11 advanced cases LDH was monitored during treatment and subsequent follow up (Figs 2, 3 & 4). In 7 cases the level returned to normal following initial treatment and subsequent investigation revealed complete resolution of disease. In 3 cases radiotherapy failed to control the disease and bring the LDH level into the normal range; all had clinical evidence of residual disease. One of these patients died, 2 responded to further treatment (chemotherapy) although one has residual disease. In one patient on follow-up a raised LDH level was noted 2 months before mediastinal adenopathy became radiologically apparent.

This study was initiated when one of our patients with Stage IV disease was found to have grossly elevated LDH levels, while other liver and cardiac enzymes were normal. It utilized established assays for enzymes and tumour markers which are readily available to the clinician at routine regional and supra regional laboratories.

Of the 7 markers evaluated in the present study on 38 patients, only LDH showed an association between raised levels and advanced disease. No attempt was made to identify any of the individual isoenzymes of which the LDH is composed.

AFP was elevated in only one case out of 30 and this patient had extensive liver involvement. It is suggested that AFP should be routinely determined in all patients with testicular tumours at first presentation. However, if the intial value is normal and the pathological diagnosis is seminoma then subsequent estimations are unhelpful.

Approximately 16% of those in whom HCG was estimated had elevated levels. All the patients with elevated levels had

FIG. 1.—Scattergram showing how the LDH level varies with stage of disease.

TABLE II.—*Relative value of tumour markers in seminoma*

| Marker | Number of patients | Median value | Range* |
|---|---|---|---|
| LDH | 38 | 388 | 116–11,440 (500) |
| AFP | 30 | <12 | <12–41 (25) |
| HCG | 31 | ⩽ 1 | ⩽1–254 (10) |
| Alanine transaminase | 37 | 25 | 7–240 (40) |
| Aspartate transaminase | 37 | 26 | 6–99 (50) |
| Alkaline phosphatase | 37 | 73 | 40–202 (92) |
| γ-Glutamyl transferase | 38 | 35 | 9–185 (118) |

* Figures in parentheses denote upper limit of normal.

advanced disease. An elevated level did not necessarily signify a poor prognosis though 2 of the 3 patients with grossly elevated levels did not respond to treatment and subsequently died. Marginal elevation of HCG may be insignificant but gross elevation ( > 100 i.u./l) may be a poor prognostic sign and an indication that the first line of treatment should be chemotherapy.

The four enzymes, GGT, Alk P, AST and ALT proved to be of no value as potential markers (Table II). Elevated levels of these enzymes were not necessarily an indication of advanced disease.

LDH has been known for many years to be elevated in patients with metastatic disease (Hill & Levi, 1954). It is also elevated in patients with viral hepatitis, cirrhosis, biliary tract disease and myocardial infarction. As a nonspecific indicator of bulk disease, LDH has been studied in patients with colorectal cancer (Beck *et al.*, 1979), gastric cancer (Carda-Abella *et al.*, 1978), uterine cancer (Marshall *et al.*, 1979) and testicular tumours.

FIG. 2.—LDH levels of 3 patients with Stage IIB disease during and after radiotherapy to the abdomen.

FIG. 3—LDH levels of patients with advanced disease during and after treatment.

In the majority of advanced cases LDH was estimated regularly during treatment and thereafter on follow-up. In 4 of the 5 cases with Stage IIB disease the LDH level returned to normal during treatment; the results of 3 of these are shown in Fig. 2. The levels have remained normal after completion of treatment. In one case in which the patient had IIB disease (Fig. 3), the level fell initially with abdominal X-ray treatment but did not return to normal. It was assumed that the patient had residual disease and a supraclavicular node was found at first follow-up. Combination chemotherapy was initiated and subsequent assessment has shown complete remission with return to normal of the LDH level.

Two patients with advanced disease (Fig. 3) did not respond to treatment. In one case XRT was stopped and chemo-

FIG. 4.—LDH levels of patients with advanced disease during and after treatment.

therapy started. Initially the patient responded and the LDH level fell. However, the tumour started to regrow before the next course of chemotherapy was given. In retrospect the time interval between courses of treatment should probably have been shortened.

This study thus illustrates that LDH levels can be readily monitored during treatment and follow-up. A return of elevated levels to normal is a useful indicator of response treatment. Continued elevation indicates the presence of residual disease. A return to an elevated level at a later time would indicate a late recurrence.

LDH as measured in routine biochemistry laboratories is made up of 5 isoenzymes—the percentage of each isoenzyme may vary from patient to patient depending upon the source of the majority of the enzyme. Blanco & Linkman (1963) reported a unique form of LDH in post-pubertal testes and sperm. Goldberg (1963) reported that the LDH-X of Blanco & Linkman was similar to LDH IV. Despite the reports of high levels of LDH IV/LDH-X in normal adult testes, Wampler & Hayra (1977) reported that LDH-1 was elevated in 2 patients with testicular tumours. Marshall *et al.* (1979) and Carda-Abella *et al.* (1978) have recorded changes in isoenzyme patterns in tumours arising at other sites. The 2 groups have suggested that the shift may be due to changes in metabolism in the tumour cells, possibly due to reduction in available oxygen in bulky tumours. Variations in amounts of specific isoenzymes may therefore only be an indication of bulky tumours and not an indication of the total tumour mass.

We would like to thank Dr P. M. Wilkinson for allowing us to report the patients treated with chemotherapy, Mrs V. Kelly and Miss C. Hunter for typing the manuscript and Mr Schofield and Staff of Medical Illustration for preparing the figures. The LDH assays were kindly performed by Dr Gowland and his staff at Withington Hospital, Manchester.

### REFERENCES

BECK, P. R., BELFIELD, A., SPOONER, R. J., BLUMGART, L. H. & WARD, C. B. (1979) Serum enzymes in colorectal cancer. *Cancer*, **43**, 1772.

BLANCO, A. & LINKMAN, W. H. (1963) Lactate dehydrogenase in human testes. *Science*, **139**, 601.

BRINDLEY, C. O. & FRANCIS, F. L. (1963) Serum lactic dehydrogenase and glutamic-oxaloacetic transaminase correlations with measurements of tumour masses during therapy. *Cancer Res.*, **23**, 112.

CARDA-ABELLA, P., PERCY-CUADRADO, S. & MATE-JIMENCY, J. (1978) LDH isoenzyme patterns in human gastric mucosa with precancerous changes. *Cancer*, **42**, 490.

GIBB, R. (1960) Seminoma of the testis. *Proc. R. Soc. Med.*, **53**, 235.

GOLDBERG, E. (1963) Lactic and malic dehydrogenases in human sperm. *Science*, **139**, 602.

GOLDMAN, R. D., KAPLAN, M. O. & HALL, T. C. (1964) Lactic dehydrogenase in human neoplastic tissues. *Cancer Ces.*, **24**, 389.

HILL, B. R. & LEVI, C. (1954) Elevation of serum component in neoplastic disease. *Cancer Res.*, **14**, 513.

MARSHALL, M. J., NEAL, F. E. & GOLDBERG, D. M. (1979) Isoenzymes of herokinase, 6-phosphogluconate dehydrogenase, phosphoglucomutase, and lactate dehydrogenase in uterine cancer. *Br. J. Cancer*, **40**, 380.

WAMPLER, G. L. & HAYRA, T. (1977) Use of LDH isoenzyme I as a serum marker for seminoma. *Proc. A.S.C.O.*, **18**, 339.


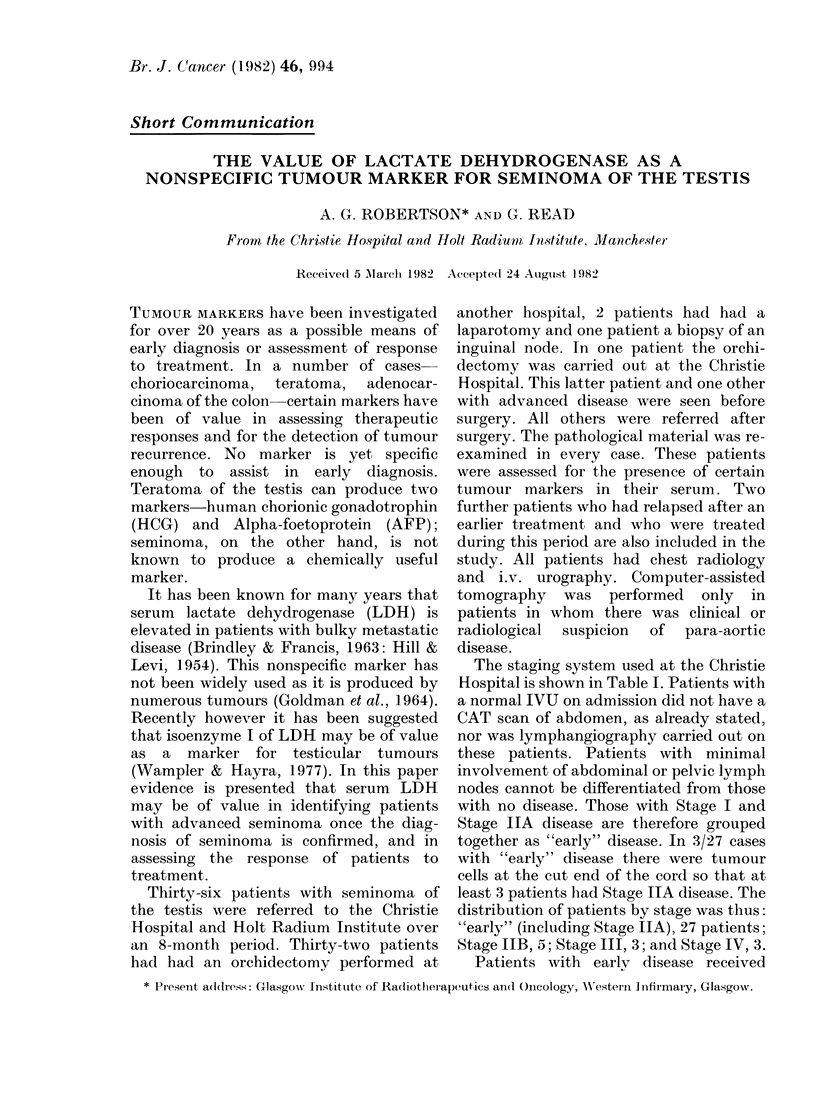

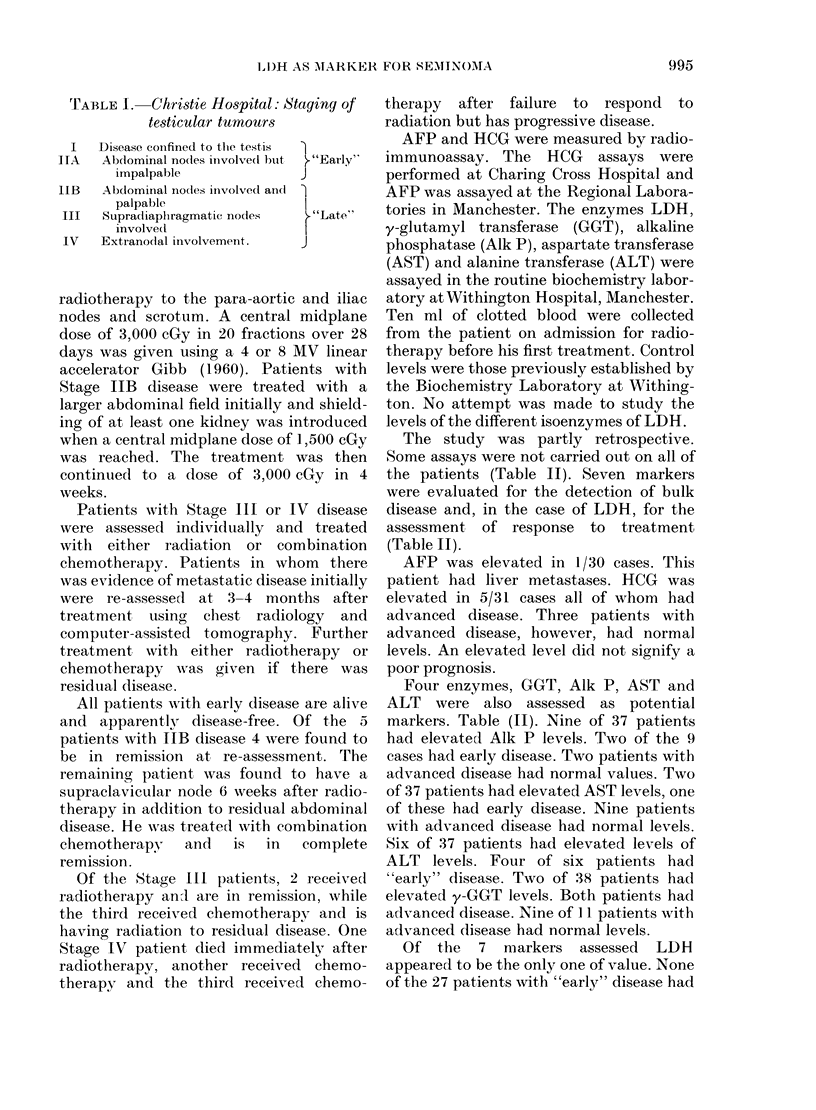

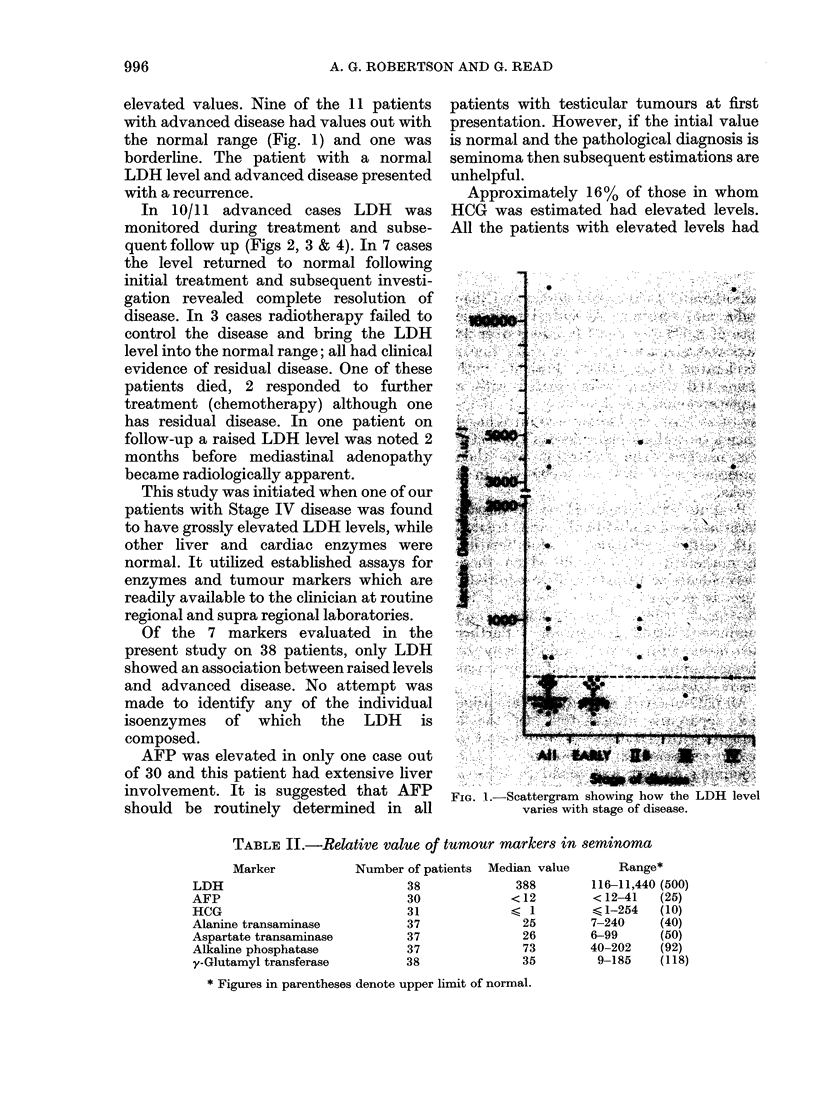

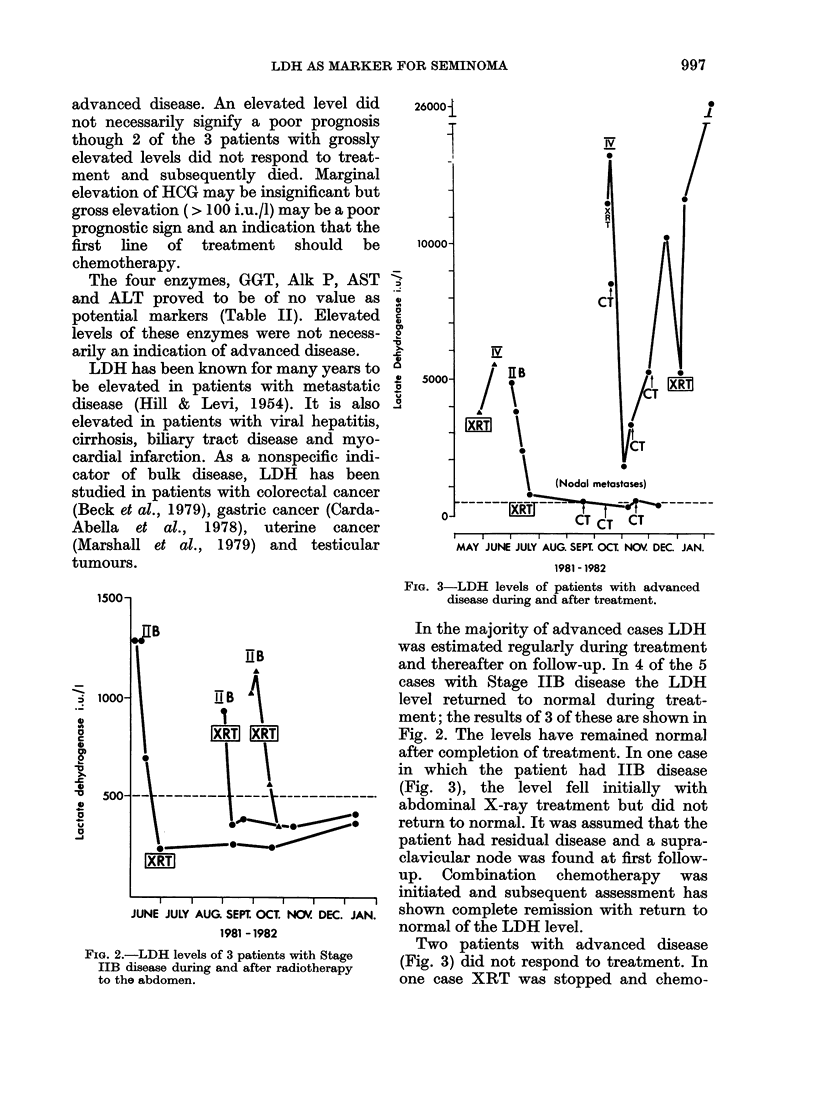

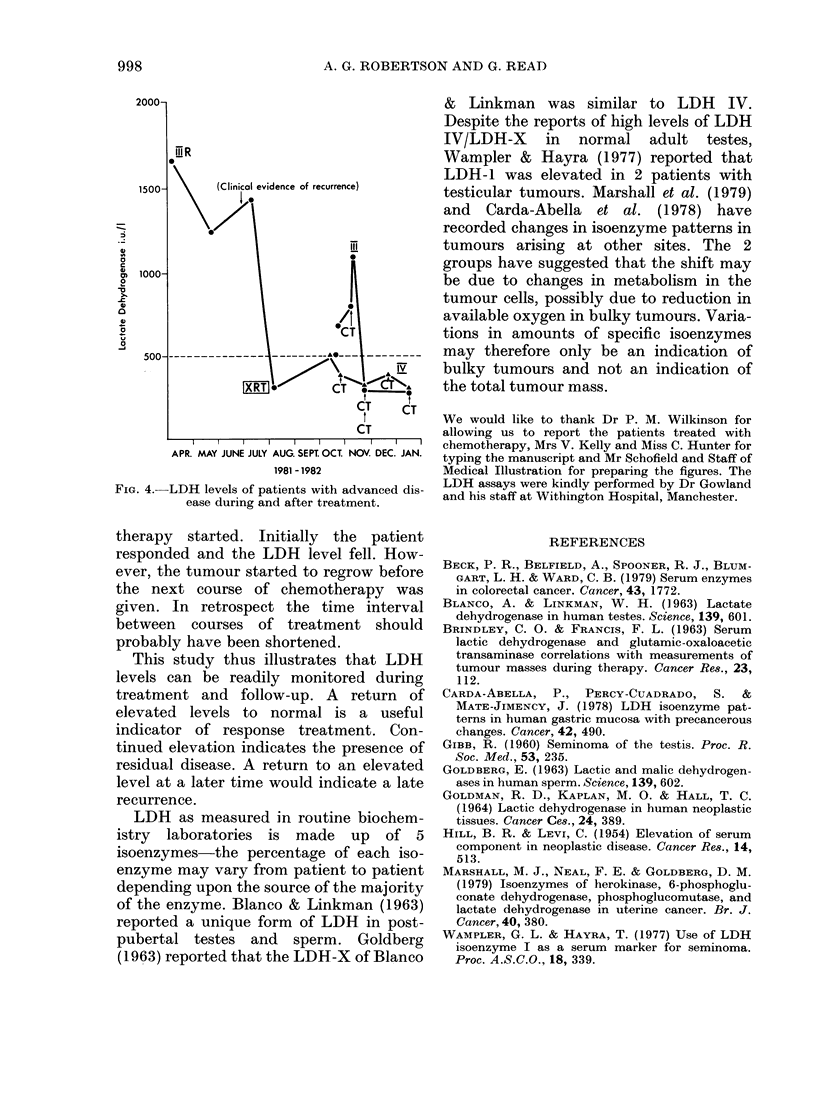


## References

[OCR_00613] BRINDLEY C. O., FRANCIS F. L. (1963). Serum lactic dehydrogenase and glutamic-oxaloacetic transminase correlations with measurements of tumor masses during therapy.. Cancer Res.

[OCR_00607] Beck P. R., Belfield A., Spooner R. J., Blumgart L. H., Wood C. B. (1979). Serum enzymes in colorectal cancer.. Cancer.

[OCR_00620] Carda-Abella P., Perez-Cuadrado M., Mate-Jimenez J. (1978). LDH isoenzyme patterns in human gastric mucosa with precancerous changes.. Cancer.

[OCR_00626] GIBB R. (1960). Some clinical aspects of megavoltage. Seminoma of the testis.. Proc R Soc Med.

[OCR_00634] GOLDMAN R. D., KAPLAN N. O., HALL T. C. (1964). LACTIC DEHYDROGENASE IN HUMAN NEOPLASTIC TISSUES.. Cancer Res.

[OCR_00630] Goldberg E. (1963). Lactic and Malic Dehydrogenases in Human Spermatozoa.. Science.

[OCR_00639] HILL B. R., LEVI C. (1954). Elevation of a serum component in neoplastic disease.. Cancer Res.

[OCR_00644] Marshall M. J., Neal F. E., Goldberg D. M. (1979). Isoenzymes of hexokinase, 6-phosphogluconate dehydrogenase, phosphoglucomutase and lactate dehydrogenase in uterine cancer.. Br J Cancer.

